# Circulating autoreactive proteinase 3^+^ B cells and tolerance checkpoints in ANCA-associated vasculitis

**DOI:** 10.1172/jci.insight.150999

**Published:** 2021-11-22

**Authors:** Alvise Berti, Sophie Hillion, Amber M. Hummel, Young Min Son, Nedra Chriti, Tobias Peikert, Eva M. Carmona, Wayel H. Abdulahad, Peter Heeringa, Kristina M. Harris, E. William St. Clair, Paul Brunetta, Fernando C. Fervenza, Carol A. Langford, Cees G.M. Kallenberg, Peter A. Merkel, Paul A. Monach, Philip Seo, Robert F. Spiera, John H. Stone, Guido Grandi, Jie Sun, Jacques-Olivier Pers, Ulrich Specks, Divi Cornec, for the RAVE-ITN Research Group

**Affiliations:** 1 Thoracic Disease Research Unit, Division of Pulmonary and Critical Care Medicine, Mayo Clinic, Rochester, Minnesota, USA.; 2 Rheumatology Unit, S. Chiara Regional Hospital and Department of CIBIO, University of Trento, Trento, Italy.; 3 INSERM UMR1227, Lymphocytes B et Autoimmunité, University of Brest, CHRU Brest, Brest, France.; 4 Department of Immunology, Mayo Clinic, Rochester, Minnesota, USA.; 5 Department of Rheumatology and Clinical Immunology and Department of Pathology and Medical Biology, University of Groningen, Groningen, Netherlands.; 6 Immune Tolerance Network, Bethesda, Maryland, USA.; 7 Division of Rheumatology, Duke University Medical Center, Durham, North Carolina, USA.; 8 Genentech Inc., South San Francisco, California, USA.; 9 Division of Nephrology and Hypertension, Mayo Clinic, Rochester, Minnesota, USA.; 10 Center for Vasculitis Care and Research, Department of Rheumatic and Immunologic Diseases, Cleveland Clinic, Cleveland, Ohio, USA.; 11 Division of Rheumatology, Department of Medicine, Division of Clinical Epidemiology, Department of Biostatistics, Epidemiology, and Informatics, University of Pennsylvania, Philadelphia, Pennsylvania, USA.; 12 Brigham and Women’s Hospital and VA Boston Healthcare System, Boston Massachusetts, USA.; 13 Division of Rheumatology, Johns Hopkins University, Baltimore, Maryland, USA.; 14 Vasculitis & Scleroderma Program, Hospital for Special Surgery, New York, New York, USA.; 15 Massachusetts General Hospital, Boston, Massachusetts, USA.; 16 The Rituximab for ANCA-Associated Vasculitis-Immune Tolerance Network (RAVE-ITN) Research Group is detailed in Supplemental Acknowledgments.

**Keywords:** Autoimmunity, Immunology, Autoimmune diseases, Vasculitis

## Abstract

**BACKGROUND:**

Little is known about the autoreactive B cells in antineutrophil cytoplasmic antibody–associated (ANCA-associated) vasculitis (AAV). We aimed to investigate tolerance checkpoints of circulating antigen-specific proteinase 3–reactive (PR3^+^) B cells.

**METHODS:**

Multicolor flow cytometry in combination with bioinformatics and functional in vitro studies were performed on baseline samples of PBMCs from 154 well-characterized participants of the RAVE trial (NCT00104299) with severely active PR3-AAV and myeloperoxidase-AAV (MPO-AAV) and 27 healthy controls (HCs). Clinical data and outcomes from the trial were correlated with PR3^+^ B cells (total and subsets).

**RESULTS:**

The frequency of PR3^+^ B cells among circulating B cells was higher in participants with PR3-AAV (4.77% median [IQR, 3.98%–6.01%]) than in participants with MPO-AAV (3.16% median [IQR, 2.51%–5.22%]) and participants with AAV compared with HCs (1.67% median [IQR, 1.27%–2.16%], *P* < 0.001 for all comparisons), implying a defective central tolerance checkpoint in patients with AAV. Only PBMCs from participants with PR3-AAV contained PR3^+^ B cells capable of secreting PR3-ANCA IgG in vitro, proving they were functionally distinct from those of participants with MPO-AAV and HCs. Unsupervised clustering identified subtle subsets of atypical autoreactive PR3^+^ memory B cells accumulating through the maturation process in patients with PR3-AAV. PR3^+^ B cells were enriched in the memory B cell compartment of participants with PR3-AAV and were associated with higher serum CXCL13 levels, suggesting an increased germinal center activity. PR3^+^ B cells correlated with systemic inflammation (C-reactive protein and erythrocyte sedimentation rate, *P* < 0.05) and complete remission (*P* < 0.001).

**CONCLUSION:**

This study suggests the presence of defective central antigen-independent and peripheral antigen-dependent checkpoints in patients with PR3-AAV, elucidating the selection process of autoreactive B cells.

**Trial registration:**

ClinicalTrials.gov NCT00104299.

**Funding:**

The Vasculitis Foundation, the National Institute of Allergy and Infectious Diseases of the NIH, and the Mayo Foundation for Education and Research.

## Introduction

The systemic autoimmune diseases granulomatosis with polyangiitis and microscopic polyangiitis are small vessel vasculitis syndromes associated with circulating antineutrophil cytoplasmic antibodies (ANCA), which target either proteinase 3 (PR3) or myeloperoxidase (MPO) (1). Distinguishing the different types of ANCA-associated vasculitis (AAV) by autoantigen specificity rather than clinicopathologic factors has been proposed for classification purposes because the presence of PR3-ANCA versus MPO-ANCA conveys unique biological and clinically useful information (2, 3).

In healthy individuals, a large fraction of immature B cells are autoreactive, but autoreactive cells are progressively eliminated by central and peripheral tolerance checkpoints during the B cell maturation and selection process (4–7). Central checkpoint mechanisms control the survival of the cells during their maturation from bone marrow–residing immature B cells to circulating transitional B cells; they may be studied by analyzing the proportion of autoreactive B cells among blood transitional B cells. Peripheral tolerance checkpoints control the maturation of B cells from the transitional to the naive mature stages and then during their functional orientation toward memory B cells and antibody-secreting cells; they may be studied by comparing the proportions of autoreactive B cells among the different circulating B cell subsets (4–7). Normal tolerance mechanisms are thought to be defective in patients with AAV (8). Yet, little is known about how autoreactive B cells escape tolerance checkpoints leading to ANCA production. The presence of circulating IgG-secreting cells and their B cell precursors bearing a B cell receptor (BCR) specific for PR3 or MPO has been postulated in AAV, but investigation of these cells has been limited by their low frequency in peripheral blood (4, 5). Furthermore, in comparison to other autoimmune diseases (6, 9–11), very few studies have characterized ANCAs reconstituted from human B cells (12, 13), and valid animal models of AAV are only available for MPO-AAV (14–17).

We previously developed an original method to enumerate PR3-reactive B cells (PR3^+^ B cells) by flow cytometry and observed a higher frequency of circulating PR3^+^ B cells that were enriched within memory B cells in a small cohort of patients with PR3-AAV as compared with healthy controls (HCs) (18).

The present study was designed to (a) confirm and expand our preliminary observations in a large, well-characterized cohort of patients with PR3-AAV as compared with patients with MPO-AAV as disease controls and HCs; (b) elucidate the mechanisms of the differentiation process leading to mature PR3^+^ B cell selection in patients with PR3-AAV during the active phase of the disease, as compared with patients with MPO-AAV and HCs; and (c) investigate the relationship between the frequency of PR3^+^ B cell subsets and the clinical and biological features of PR3-AAV.

## Results

### Perturbations of circulating B cell subsets are similar in patients with PR3-AAV and MPO-AAV.

**Figure 1:**
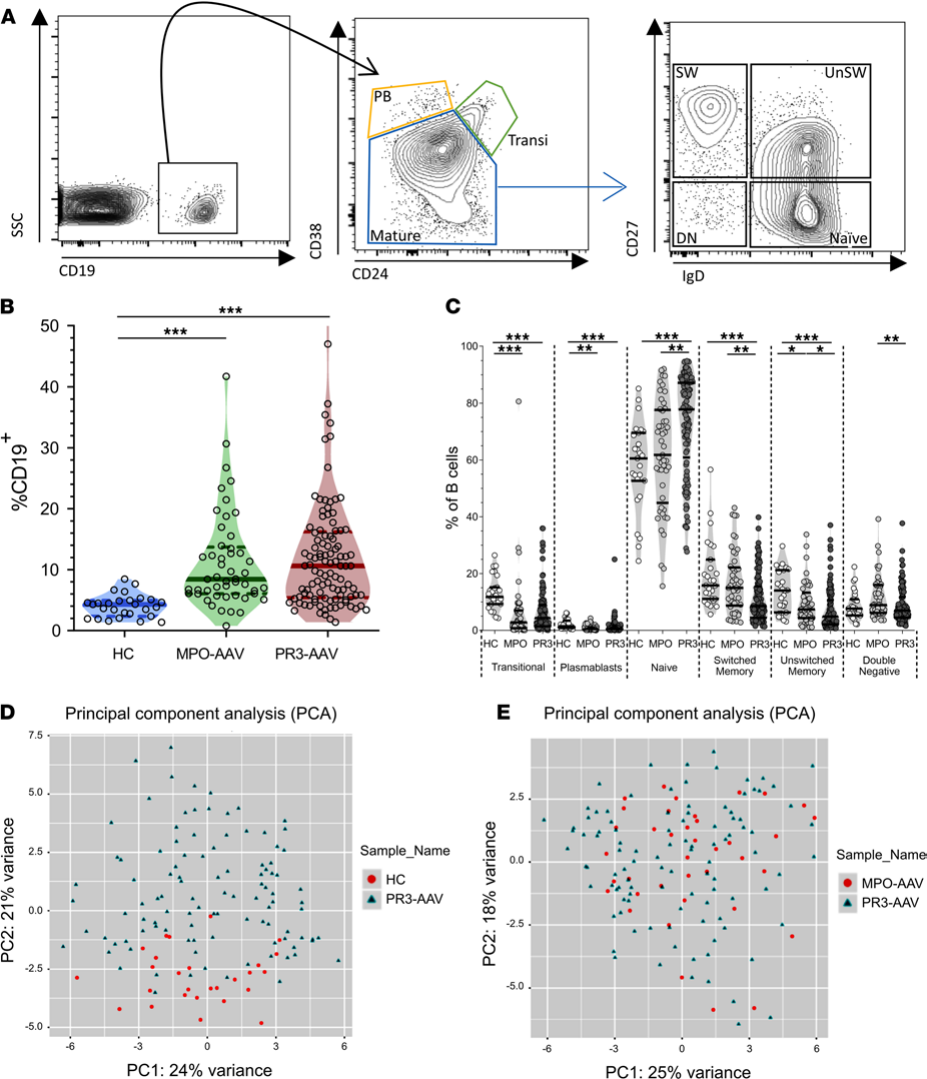
Circulating B cells in patients with PR3-AAV and MPO-AAV and HCs. Gating strategy used to define B cell subsets (**A**). CD19^+^ cells were first categorized based on CD24 and CD38 expression, in transitional B cells (transi, CD24^hi^CD38^hi^), plasmablasts (PB, CD24^–^CD38^hi^), and mature B cells (CD24^+^CD38^+^). Mature B cells were further classified into 4 populations: naive (CD27^–^IgD^+^), unswitched memory (UnSW; CD27^+^IgD^+^), switched memory (SW; CD27^+^IgD^–^), and double negative (DN, CD27^–^IgD^–^). B cell frequency and subset distribution were overall similar in patients with PR3-AAV (*n* = 105) and MPO-AAV (*n* = 49) but different compared with HCs (*n* = 27) (**B** and **C**). Principal component analysis of the 200 B cell clusters obtained with spanning-tree progression analysis of density-normalized events (SPADE) representing HCs and PR3-AAV trial participants (**D**) and participants with MPO-AAV and PR3-AAV (**E**). Data represent median (25%–75% IQR). Multiple comparisons among more than 2 groups were performed with Kruskal-Wallis test. *P < 0.05, **P < 0.01, ***P < 0.001 after correction for FDR with Benjamini and Hochberg test.

The characteristics of the participants are shown in Supplemental Table 1 (supplemental material available online with this article; https://doi.org/10.1172/jci.insight.150999DS1). We observed that B cell homeostasis was abnormal in patients with AAV compared with HCs (gating strategy, Figure 1A; percentages of total B cells and B cell subsets, Figure 1, B and C), confirming previous studies (19–23). The absolute counts of both participants with PR3-AAV and MPO-AAV showed higher numbers of circulating B cells and higher numbers of naive and double-negative (DN) B cells than HCs (Supplemental Figure 1, A–C).

We further dissected the B cell pool using the spanning-tree progression analysis of density-normalized events (SPADE) algorithm (Supplemental Figure 1D). Overall, the B cell clusters segregated participants with PR3-AAV from HCs when directly compared (principal component analysis, Figure 1D; unsupervised hierarchical clustering heatmap, Supplemental Figure 1E) but not from participants with MPO-AAV, meaning that the 2 groups of patients display similar disturbances in B cell homeostasis compared with HCs (Figure 1E and Supplemental Figure 1F).

### Circulating PR3^+^ B cells are higher in patients with PR3-AAV than in controls.

**Figure 2:**
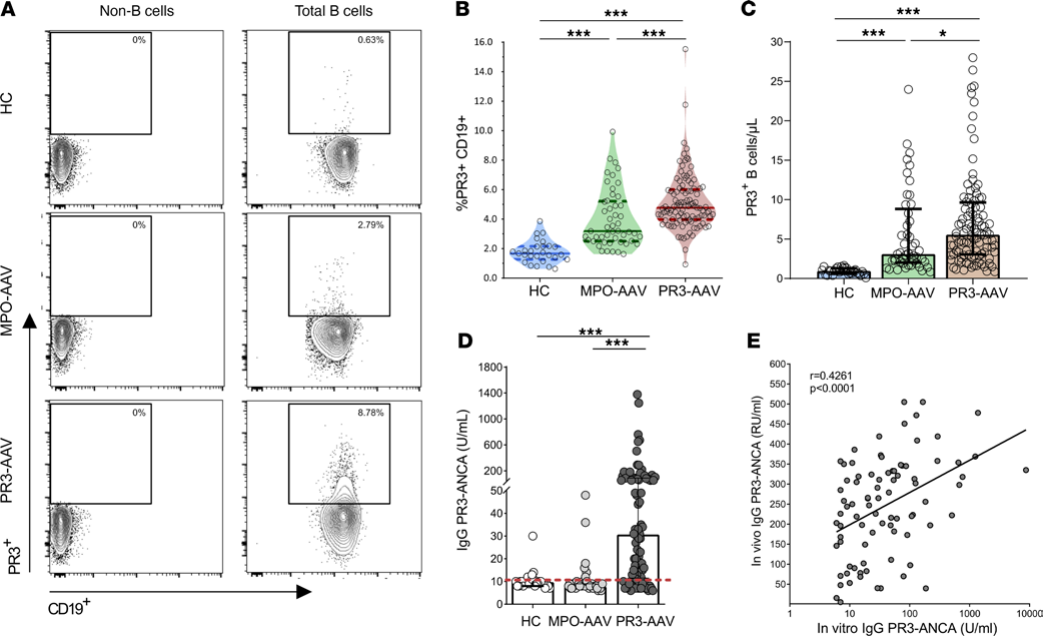
Circulating PR3^+^ B cells and PR3-ANCA production in patients with PR3-AAV and MPO-AAV and HCs. Representative examples of the gating of PR3^+^ B cells among total CD19^+^ cells in a patient with PR3-AAV, a patient with MPO-AAV, and a HC (**A**). PR3^+^ B cell frequency and count were increased in patients with PR3-AAV (*n* = 105) compared with patients with MPO-AAV (*n* = 49) and HCs (*n* = 27) (**B** and **C**). PBMCs were cultured to promote differentiation into antibody-secreting cells, after which PR3-ANCA secretion was analyzed by means of a Phadia ImmunoCAP 250 analyzer (**D**). Only patients with PR3-AAV can produce PR3-ANCA IgG in vitro. Correlation of circulating (in vivo) PR3-ANCA IgG with secreted (in vitro) PR3-ANCA IgG in patients with PR3-AAV (**E**). Data represent median (25%–75% IQR). Multiple comparisons among more than 2 groups were performed with Kruskal-Wallis test. *P < 0.05, ***P < 0.001 after correction for FDR with Benjamini and Hochberg test.

PR3^+^ B cells were detected in PBMCs from patients with PR3-AAV and MPO-AAV as well as HCs (representative plots, Figure 2A). Patients with PR3-AAV had higher frequencies and absolute numbers of PR3^+^ B cells compared with patients with MPO-AAV and HCs (median [25%–75% IQR], PR3-AAV, 4.77% [3.98%–6.01%] versus MPO-AAV, 3.19% [2.51%–5.22%] versus HC, 1.67% [1.27%–2.16%], *P* < 0.001 for all comparisons; PR3-AAV, 5.55 cells/μl [3.09–9.64 cells/μl] versus MPO-AAV, 3.09 cells/μl [2.02–8.81 cells/μl], *P* < 0.05, and MPO-AAV versus HC, 0.95 cells/μl [0.58–1.31 cells/μl] cells/μl, *P* < 0.001), confirming and expanding the findings from our previous report (18) (Figure 2, B and C). Notably, no significant effect of glucocorticoids on the levels and percentages of lymphocytes, B cells, PR3^+^ B cells, and other T cell specific subsets was observed (Supplemental Table 2).

### PBMCs from patients with PR3-AAV contain PR3^+^ B cells capable of secreting PR3-ANCA IgG in vitro.

Supernatants from PBMC cultures from patients with PR3-AAV contained significantly higher levels of anti-PR3 IgG than those from patients with MPO-AAV and HCs (*P* < 0.001, Figure 2D), showing that PR3^+^ B cells from patients with PR3-AAV are functionally distinct from their counterparts from patients with MPO-AAV and HCs.

Then, we sought to assess whether PR3^+^ B cells that can be detected among PBMCs by FACS are responsible for the in vitro secretion of anti-PR3 immunoglobulin (Ig). We measured the frequency of PR3^+^-secreting cells (IgM and IgG) by ELISPOT after stimulation. After sorting PR3^+^ and PR3^–^ B cells from B cell–enriched PBMCs of HCs (Supplemental Figure 2, A and B), we found that circulating PR3^+^ B cells from HCs are able to produce PR3-ANCA IgM but not PR3-ANCA IgG. Furthermore, we could not detect any PR3-specific antibody secretion in PR3^–^ cells, demonstrating that our detection method ensures a full recovery of circulating PR3-specific B cells within the PR3^+^ B cell pool as detected by FACS.

The significant correlation between anti-PR3 IgG levels from in vitro cultures and in vivo serum levels in participants with PR3-AAV (Figure 2E and Supplemental Figure 2C) suggests that circulating PR3^+^ B cells from patients with PR3-ANCA can serve as bona fide precursors of PR3-ANCA IgG-secreting cells in vivo.

### Autoreactive PR3^+^ memory B cells accumulate through the maturation process only in patients with PR3-AAV.

**Figure 3:**
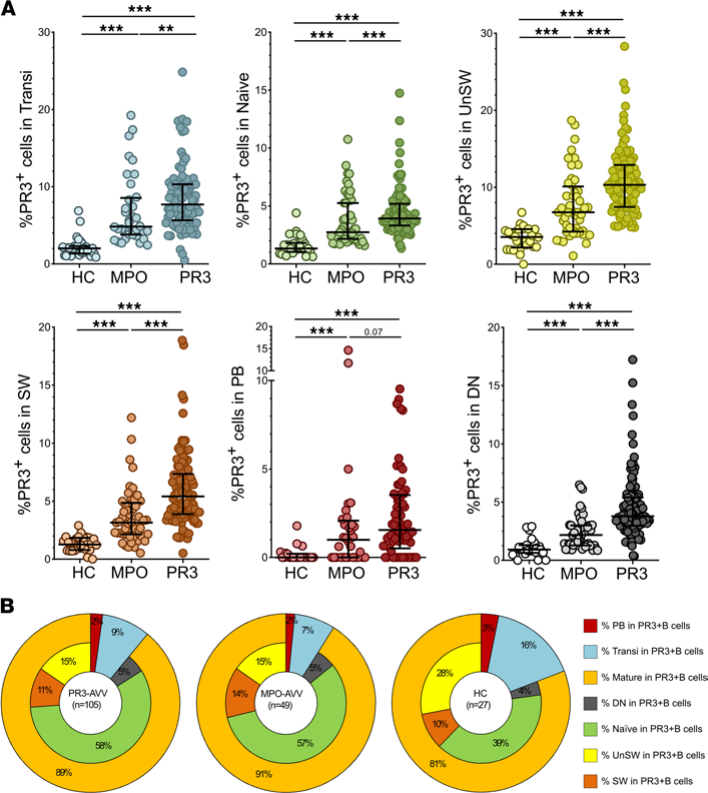
Frequency of PR3^+^ B cells within each B cell subset. Scatter plots depicting the frequency of PR3^+^ B cells within each B cell subset in HCs (*n* = 27), patients with MPO-AAV (*n* = 49), and patients with PR3-AAV (*n* = 105) (**A**). B cell subset distribution within PR3^+^ pool in patients with PR3-AAV and MPO-AAV and HCs (**B**). Data represent median (25%–75% IQR). Multiple comparisons among more than 2 groups were performed with Kruskal-Wallis test. **P < 0.01, ***P < 0.001 after correction for FDR with Benjamini and Hochberg test.

The frequency of PR3^+^ B cells within each of the different B cell subsets was higher in patients with PR3-AAV than in patients with MPO-AAV, and in patients with AAV overall compared with HCs, implying a defective central tolerance checkpoint in patients with AAV compared with HCs (Figure 3A, and representative plots, Supplemental Figure 3). B cell subsets within the PR3^+^ B cell pool were similarly distributed in patients with PR3-AAV and MPO-AAV and HCs, except for higher levels of mature B cells in patients with PR3-AAV and MPO-AAV compared with HCs (*P* < 0.001) (Figure 3B).

**Figure 4:**
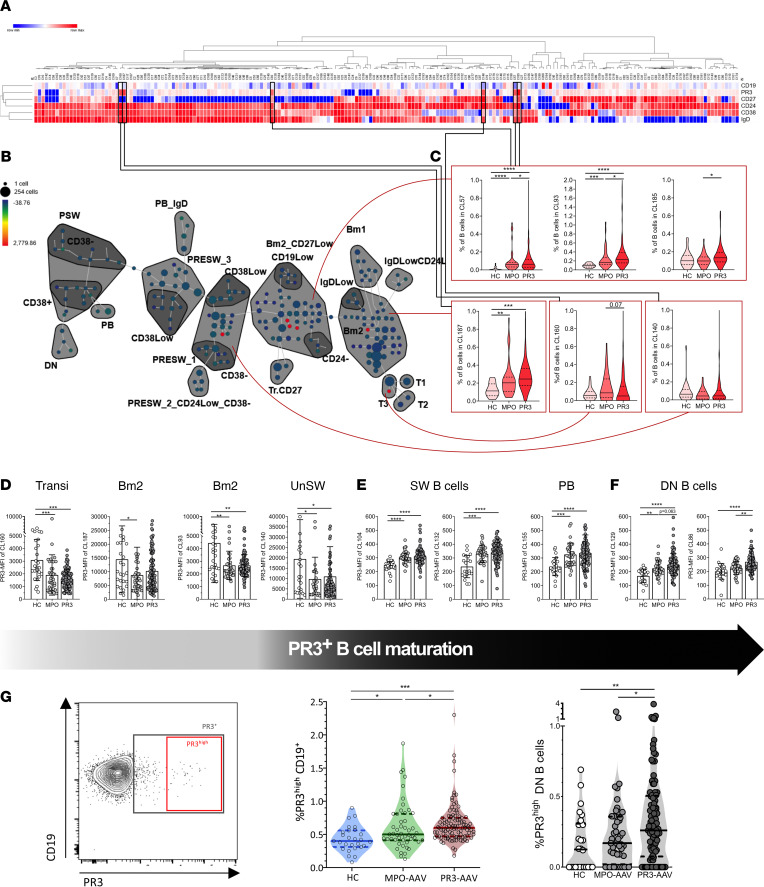
Selected PR3-reactive B cell clusters are significantly more represented or activated in patients with PR3-AAV. Six of 200 clusters showed a stable expression of PR3 on the membrane of the B cells (**A**). A processed SPADE explanatory image of 1 patients with PR3-AAV, showing the most relevant clusters grouped by conventional subsets (**B**). In red, the six clusters of B cells with a stable increased reactivity for PR3 across all the samples, and their frequencies (**C**). The MFI of 4 of these 6 clusters is reduced in AAV compared with HCs (**D**). Additional 5 clusters with significantly varied MFI between HCs and patients with AAV: the MFIs of clusters within the SW memory and PB compartments are increased in patients with AAV compared with HCs (**E**), and 2 clusters within the DN showed a relative MFI increase in patients with PR3-AAV compared with patients with MPO-AAV and HCs (**F**). A more conservative gating approach (PR3^hi^) (**G**, left), showing the increase of PR3^hi^ B cells in patients with PR3-AAV compared with those with MPO-AAV and HCs (**G**, middle). Among B cell subsets, PR3^hi^ B cells were significantly increased only in the DN subset in participants with PR3-AAV compared with participants with MPO-AAV and HCs (**G**, right). Each point represents the frequency in an individual; horizontal lines show the median with 25%–75% IQR; each histogram represents mean ± SD. Multiple comparisons among more than 2 groups were performed with 1-way ANOVA or Kruskal-Wallis test, where appropriate. *P < 0.05, **P < 0.01, ***P < 0.001, ****P < 0.0001 after correction for FDR with Benjamini and Hochberg test. MFI, mean fluorescence intensity.

In order to better understand whether rare subsets of PR3^+^ B cells were specifically enriched in patients with PR3-AAV compared with controls, we identified PR3-reactive B cell clusters defined by the SPADE algorithm significantly more represented or activated in patients with PR3-AAV. Among the 200 B cell nodes, 6 clusters of cells displayed a stable increased reactivity for PR3 across all the samples in HCs and participants with MPO-AAV and PR3-AAV (Figure 4, A and B, and phenotypic characterization, Supplemental Table 3). Among these 6 stable PR3^+^ clusters, we observed a significant enrichment of PR3^+^ cells in CL57, CL93, CL160, and CL185 (Bm2/naive and transitional clusters) in patients with PR3-AAV compared with patients with MPO-AAV and HCs (Figure 4C), confirming a deficient central checkpoint (controlling the maturation of immature B cells toward transitional and naive mature B cells).

Investigation of the mean fluorescence intensity (MFI) of the PR3 BCR in these transitional/naive clusters (Figure 4D and Supplemental Figure 4A) revealed a marked reduction in CL160, CL187, CL93, and CL140 in patients with AAV, a hallmark of chronically activated or anergic cells. In contrast, the PR3 BCR MFI was significantly higher in 5 other clusters (CL86, CL129, CL104, CL132, CL155), belonging to the memory compartments, suggesting the presence of defective peripheral checkpoints in AAV. Clusters CL104 and CL132 (corresponding to switched activated memory cells) and CL155 (corresponding to plasmablasts [PB]) were increased in patients with AAV with no significant differences between patients with PR3-AAV and MPO-AAV (Figure 4E); a PR3-specific defect was identified in the clusters CL86 and CL129 (corresponding to the DN IgD^–^CD27^–^CD38^–^ subset) only in patients with PR3-AAV (Figure 4F).

We therefore repeated the manual gating analysis using a more conservative strategy to identify PR3^+^ B cells with the highest reactivity (PR3^hi^) (gating strategy, Figure 4G, left) and observed that similar to the frequency of total PR3^+^ B cells (Figure 1B), the frequency of PR3^hi^ B cells was significantly increased in patients with PR3-AAV compared with patients with MPO-AAV and HCs (Figure 4G, middle). Consistent with the unsupervised cluster analyses, enrichment of PR3^hi^ B cells among the peripheral pool of DN B cells distinguished patients with PR3-AAV from both patients with MPO-AAV and HCs (Figure 4G, right, and Supplemental Figure 4B).

### Selection of mature PR3^+^ B cells and determinants of the maturation of autoreactive B cells.

**Figure 5:**
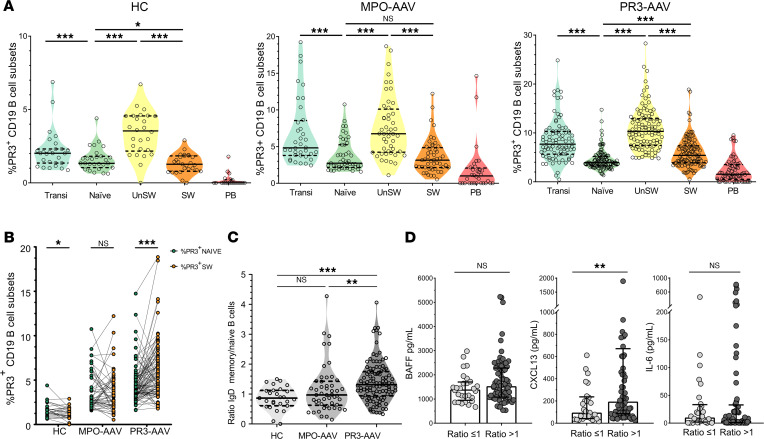
Maturation of PR3^+^ B cells among different participant groups. Scatter plots depicting the different frequency of PR3^+^ B cells within each B cell subset through the maturation process in HCs (*n* = 27), participants with MPO-AAV (*n* = 49), and participants with PR3-AAV (*n* = 105). The multiple comparisons on B cell maturation were analyzed by using mixed-effects modeling. (**A**). Paired comparisons between PR3^+^ naive and SW mature PR3^+^ B cells, showing the enrichment of memory B cells in participants with PR3-AAV, but not in participants with MPO-AAV and HCs (**B**). Ratio between the frequency of PR3^+^ B cells among IgD^–^ switched memory and the frequency of PR3^+^ B cells among naive B cells (**C**). Each point represents the frequency in an individual; horizontal lines show the median with 25%–75% IQR. Multiple comparisons among more than 2 groups were performed with Kruskal-Wallis test. Selected cytokine (BAFF, CXCL13, IL-6) levels by SW memory/naive PR3^+^ B cell ratio in patients with PR3-AAV. *P* values were determined by 2-tailed Mann-Whitney test (**D**). *P < 0.05, **P < 0.01, ***P < 0.001 after correction for FDR with Benjamini and Hochberg test.

When comparing the frequencies of PR3^+^ B cells in each B cell subset within each disease group, we observed a significant decrease in the frequencies of PR3^+^ B cells from transitional to naive, from unswitched memory (UnSW) to switched memory (SW), and from SW to PB in all the groups, illustrating the different layers of control of the peripheral checkpoints (Figure 5A). Participants with PR3-AAV showed a higher frequency of PR3^+^ B cells in the SW compartment compared with the naive one, while patients with MPO-AAV and HCs did not (Figure 5B).

To illustrate the differential enrichment of PR3^+^ B cells during the maturation process, we computed a ratio of the frequency of PR3^+^ B cells within SW B cells over the frequency of PR3^+^ B cells within naive B cells. The median ratio was less than 1 in HCs and patients with MPO-AAV but was more than 1 in participants with PR3-AAV (Figure 5C), with approximately one-third of HCs, half of the participants with MPO-AAVs and almost three-quarters of the participants with PR3-AAV with a ratio of more than 1 (Supplemental Figure 5A).

In addition to having higher proportions of PR3^+^ SW B cells relative to the proportions of PR3^+^ naive B cells (accounting for a higher ratio), participants with PR3-AAV with a ratio of more than 1 compared with those with ratios of less than or equal to 1 had higher levels of circulating PR3^+^ PB and DN B cells and lower levels of transitional B cells, suggesting that the ratio reflects an overall higher degree of PR3^+^ B cells maturation (Table 1).

We measured the relationship between PR3^+^ B cell maturation and serum cytokine levels potentially implicated, which was similar between patients with MPO-AAV and PR3-AAV (Supplemental Figure 5B). While B cell–activating factor (BAFF) and IL-6 did not show any association with PR3^+^ B cell maturation in patients with PR3-AAV, C-X-C motif ligand 13 (CXCL13) levels were higher in those with a SW memory/naive ratio of more than 1 compared with those with a ratio of less than or equal to 1 (Figure 5D), suggesting a higher germinal center reaction (24) for participants with PR3-AAV with a SW memory/naive ratio of more than 1.

The absolute numbers of chemokine receptor 5–positive (CXCR5^+^) programmed cell death-1–positive (PD-1^+^) T follicular helper (CXCR5^+^PD-1^+^ Tfh) cells were significantly elevated in patients with PR3-AAV and MPO-AAV compared with HCs (Supplemental Figure 5, C and D), in line with observations made by others (25). Additionally, we observed that circulating Tfh count and frequency were lower in participants with PR3-AAV with a SW memory/naive ratio of more than 1 compared with those with a ratio of less than or equal to 1 (Supplemental Figure 5E). Together, these results support the activation of the germinal center machinery to promote PR3-reactive B cell maturation in participants with PR3-AAV.

### Maturation of PR3^+^ B cells and clinical manifestations in patients with PR3-AAV.

**Figure 6:**
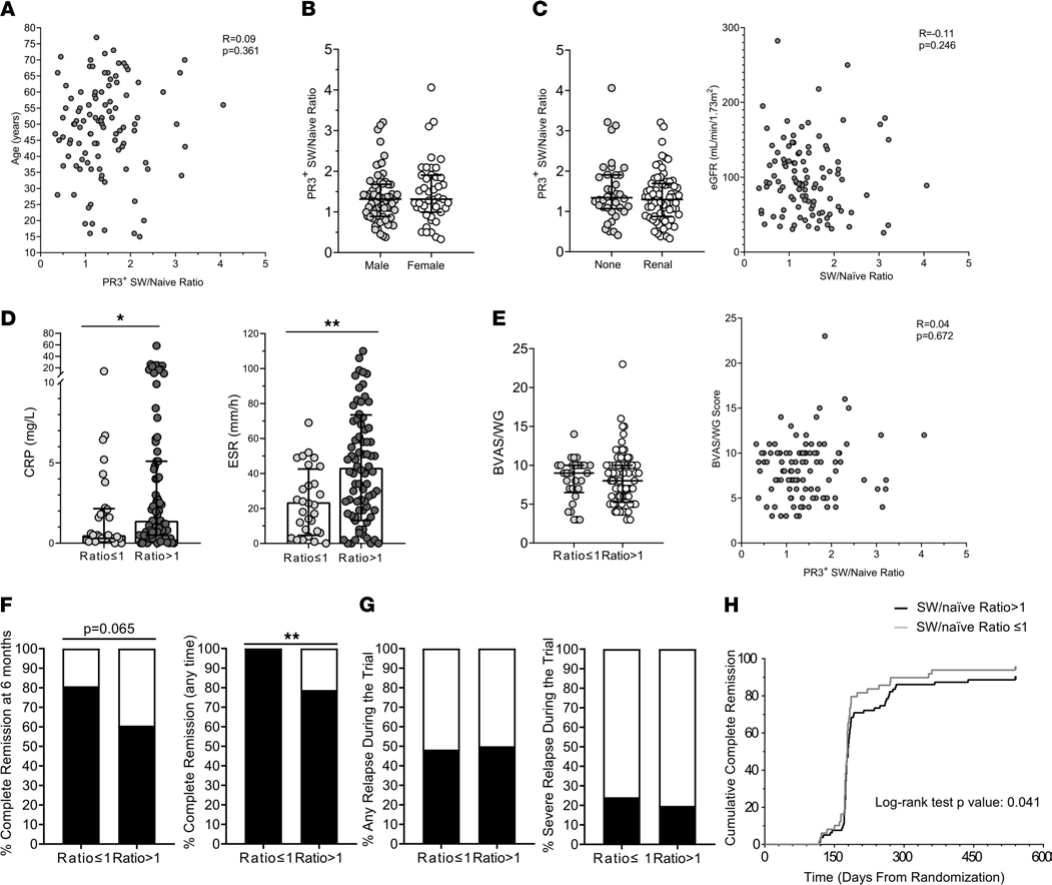
SW memory/naive PR3^+^ B cell ratio and clinical manifestations in patients with PR3-AAV. In participants with PR3-AAV (*n* = 105), the SW memory/naive PR3^+^ B cell ratio did not correlate with age (**A**), sex (**B**), or renal manifestations (**C**), but higher levels of CRP and ESR were associated with a ratio of more than 1 (**D**). The ratio did not correlate with disease activity as assessed by BVAS/WG (**E**). Associations with complete remission definitions (**F**), future relapse and severe relapse (**G**), and time-to-complete remission (**H**) are represented. When evaluating associations with remission, the patients that underwent crossover (*n* = 7) or experienced early treatment failure (*n* = 6) during the trial time were excluded from the analysis. Data represent median (25%–75% IQR), while histograms represent proportions. *P* values were determined by 2-tailed Mann-Whitney test, or Fisher’s test, where appropriate. Spearman’s test and the Kaplan-Meier method with the log-rank test were used to test correlations and time to event, respectively. *P < 0.05, **P < 0.01, ***P < 0.001.

The PR3^+^ SW memory/naive B cell ratio did not show significant correlations with clinical and demographic features (Figure 6, A–C, and Supplemental Figure 6, A–E). Participants with ratios of more than 1 had higher erythrocyte sedimentation rate (ESR) and C-reactive protein (CRP) serum levels compared with those with a ratio of less than or equal to 1 (Figure 6D), but disease activity assessed by Birmingham Vasculitis Activity Score for Wegener’s Granulomatosis (BVAS/WG) did not show any association with this ratio (Figure 6E).

Whereas participants with ratio of less than or equal to 1 tended to achieve more frequent complete remission at 6 months than those with ratios of more than 1 (80.8% vs. 60.6%, respectively, *P* = 0.0649; primary endpoint of the trial, defined as BVAS/WG = 0 and prednisone = 0), 100% of participants with ratios of less than or equal to 1 achieved complete remission at any time during the observation period compared with less than 80% of those with ratio of greater than 1 (*P* ≤ 0.001) (Figure 6F). Time-to-complete remission (Figure 6H) and time-to-first remission (BVAS/WG = 0) (Supplemental Figure 6F) were significantly shorter in participants with ratios of less than or equal to 1 compared with those with ratios of more than 1. Future relapse and severe relapse were not associated to this ratio (Figure 6G). No striking correlations between the frequency of PR3^+^ B cells and the demographic, disease activity, and major clinical features were observed (Supplemental Figure 7).

Taken together, a PR3^+^ SW memory/naive B cell ratio of more than 1 seems to be associated with higher degrees of systemic inflammation and slower response to treatment.

## Discussion

Herein, we confirm our previous findings (18) in a large well-characterized cohort of patients with AAV with active disease and show that patients with PR3-AAV have higher circulating levels of PR3^+^ B cells compared with healthy and disease controls, suggesting a general defect in B cell tolerance in patients with AAV compared with HCs. Notably, only circulating B cells from PR3-AAV secrete PR3-ANCA IgG under appropriate stimulation, reflecting their in vivo activity. While the frequency of PR3^+^ B cells decreased as B cells progress through the maturation checkpoints in patients with AAV and HCs, there appears to be a preferential enrichment of PR3^+^ B cells in the memory B cell subsets of patients with PR3-AAV. This phenomenon is accompanied by signs of germinal center activation, suggesting an antigen-specific breach in this layer of control of peripheral B cell maturation. The selection of these autoreactive B cells through the different B cell compartments results in an accumulation of autoreactive PR3^+^ memory B cells, particularly in PR3^+^ DN subsets. The association between the maturation of PR3^+^ B cells, as reflected by the IgD^–^ memory/naive ratio, with CRP, ESR, and complete remission suggests a link between autoimmunity and systemic inflammation in AAV.

Interestingly, when focusing on the frequency of PR3^+^ B cells within each cell subset, the frequency of PR3^+^ B cells was also elevated in patients with MPO-AAV compared with HCs, suggesting a defect in an early central tolerance checkpoint due to impaired selection of B cells before the transitional stage in patients with both PR3- and MPO-AAV. Self-reactive human pre- or pro-B cells are usually eliminated before reaching the naive B cell stage by receptor editing or clonal deletion, while antigen-experienced B cell populations are further excluded by induction of anergy and follicular exclusion (11, 26–29). A possible mechanism contributing to the increase of PR3^+^ B cells in patients with MPO-AAV might be a nonspecific defect in clonal anergy predisposing to autoimmunity (30), because levels of the circulating PR3 antigen are increased in patients with both PR3-AAV and MPO-AAV compared with HCs as a consequence of neutrophil activation. A possible explanation of the subsequent tolerance failure in patients with PR3-AAV, but not in patients with MPO-AAV, might be the interplay between different genetic factors and autoreactive PR3^+^ B cells. It is well documented that MPO-AAV and PR3-AAV have different HLA and non-HLA gene associations, i.e., for PR3-AAV, HLA-DP and the genes encoding for α1-antitrypsin (SERPINA1) and PR3 (PRTN3) (31). Even though both patients with MPO-AAV and PR3-AAV have increased levels of autoreactive PR3^+^ B cells, it is only those from patients with PR3-AAV that are able to maturate and produce PR3-ANCA IgG, possibly because of the genetic predisposition conferred by these genes. Beside the HLA, mutations of the genes coding for the PR3 antigen, the PR3-specific BCR (i.e., an ANCA immunoglobulin), and possibly the α1-antitrypsin inhibiting PR3 might increase the affinity of this tripartite interaction, leading to a breach in immune tolerance.

However, despite this mild increase of PR3^+^ B cells in patients with MPO-AAV, only circulating B cells of patients with PR3-AAV can produce PR3-ANCA IgGs, suggesting that only patients with PR3-AAV can provide adequate T cell help that may be instrumental to bypass anergy mechanisms.

Hierarchical B cell clustering provided evidence of qualitative differences among the different patient groups. Patients with AAV seem to display a more permissive microenvironment, leading to the emergence of autoreactive B cell clones, whereas PR3^+^ B cells progress through the maturation stages. Importantly, we identified rare subsets of atypical autoreactive PR3^+^ memory B cells accumulating through the maturation process in patients with PR3-AAV as compared with patients with MPO-AAV and HCs. A similar antigen-specific defect has recently been identified for antinuclear antibody-secreting B cells as a source of pathogenic antibodies in SLE (32). DN B cells have characteristics of memory B cells and are thought to represent a source of autoreactive antibody-secreting plasmablasts in SLE (32).

Patients with PR3-AAV showed a higher degree of maturation of PR3^+^ B cells compared with both disease and HCs, as represented by a significantly higher PR3^+^IgD^–^ memory/naive ratio and a higher frequency of participants with ratios of more than 1 compared with participants with MPO-AAV and HCs. Serum CXCL13 levels were significantly increased, and the proportion of Tfh cells was decreased in patients with PR3-AAV with a PR3^+^ ratio of more than 1 compared with those with a ratio less than or equal to 1, suggesting increased germinal center activity in patients with PR3-AAV with a higher PR3^+^IgD^–^ memory/naive ratio. B cell maturation takes place in germinal centers of secondary lymphoid tissue or in tertiary lymphoid organ structures in inflamed target tissues in patients with autoimmune diseases, where the interaction of the recruited antigen-specific B cells with cytokines and cells (i.e., activated Tfh cells) leads to expansion, differentiation, and, ultimately, positive selection of antibody-secreting B cells and memory B cells (33–36). The major limitation of the study is that lymphoid tissue from patients with AAV was not available, impairing a direct assessment of the germinal center microenvironment. Therefore, we measured selected circulating cytokines, such as CXCL13, previously shown to reflect germinal center activation in lymph nodes in clinical and preclinical models of autoimmune and infectious diseases, as well as circulating Tfh (24, 37). Altogether, these data support the maturation process of PR3^+^ B cells ultimately leading to a preferential enrichment of PR3^+^ B cells in the memory compartment of patients with PR3-AAV as compared with those of patients with MPO-AAV and HCs.

From a clinical perspective, the PR3^+^ SW memory/naive B cell ratio seems to correlate with the degree of systemic inflammation, as measured by serological markers of inflammation used in clinical practice, CRP and ESR, and by the time to achieving complete remission, but not with disease activity scored by the BVAS/WG, an instrument that iterates all disease manifestations affecting a patient within a 28-day period leading up to the date of the biological sampling. BVAS/WG has sensitivity to measure disease activity, which could guide the need for immunosuppressive treatment, but it is probably a suboptimal gauge of systemic inflammation at a specific point in time when serum, plasma, and PBMCs are collected. Overall, our findings suggest a possible link between the maturation of PR3^+^ B cells and the burden of systemic inflammation caused by AAV, an observation that requires confirmation in future clinical studies.

With this work, we elucidate for the first time to our knowledge the positive selection of autoreactive PR3^+^ B cells in PR3-AAV responsible for generating a distinct subset of mature autoreactive B cells in the peripheral circulation, which are likely the source of PR3-ANCA IgG in vivo*.* These findings begin to uncover the layers of control that are deficient in patients with AAV and that promote the development of PR3-ANCA.

## Methods

### Study population and definitions.

All available baseline cryopreserved PBMC samples from the Rituximab in ANCA-Associated Vasculitis (RAVE) trial (NCT00104299, ref. 38) were collected and used for our analysis. We analyzed 181 participants, 105 with PR3-AAV, 49 with MPO-AAV, and 27 HCs. All clinical data were obtained from the trial database. Disease activity was measured using the BVAS/WG (39).

The RAVE study (38) was a multicenter, double-blind, placebo-controlled trial that randomized 197 participants (all ANCA^+^) in a 1:1 ratio to receive either rituximab (375 mg/m^2^ intravenously each week for 4 weeks or cyclophosphamide (2 mg/kg) for 3–6 months followed by azathioprine (2 mg/kg, up to 150 mg/day).

Initially, 110 PR3-AAV trial participants and 53 participants with MPO-AAV were included in the current study, while 27 age-matched volunteers were used as HCs. A total of 9 participants were excluded from the final analysis because of depleted B cells, i.e., <10 cells/μL (*n* = 6; 4 PR3-AAV and 2 MPO-AAV) or because of artifacts/technical problems with the staining (*n* = 3; 1 from PR3-AAV, 2 from MPO-AAV). In total, we analyzed 181 participants: 105 with PR3-AAV, 49 with MPO-AAV, and 27 HCs. Disease activity was measured using the BVAS/WG (39). The primary outcome of the trial was defined as complete remission (BVAS/WG = 0 and prednisone = 0) within 6 months from randomization; other outcomes were complete remission, defined as BVAS/WG = 0 and prednisone = 0 at any time of the follow-up; complete response, defined as BVAS/WG = 0 and prednisone ≤10 mg/d; and first remission, defined as a BVAS/WG = 0, regardless of the dose of glucocorticoids. Disease relapse was defined as any new disease activity, with an increase in BVAS/WG of more than or equal to 1 point after achievement of CR. Severe relapse was defined as a BVAS/WG of more than or equal to 3 or the occurrence of at least 1 major BVAS/WG item following disease remission requiring retreatment with either rituximab or cyclophosphamide.

### Recombinant PR3 production and labeling.

A recombinant PR3 (rPR3) was expressed in an epithelial cell line as previously described (40). This variant consisted of the mature form of the protein (deletion of the N-terminal prodipeptide, allowing a mature conformational state), that was enzymatically inactive (S195A point mutated to avoid the protease activity that could digest different proteins, including immunoglobulins), and produced by stable transfection of HEK293 cells (41). This rPR3 is well recognized by PR3-ANCA from patients with AAV (42). Culture supernatants were harvested after a 48-hour starvation. Then, rPR3 was purified using a column loaded with the anti-human PR3 monoclonal antibody MCPR3-2 (43) following recommendations from the supplier (CNBr-Activated Sepharose 4 Fast Flow, GE HealthCare); it was then concentrated and quantified by Coomassie Plus (Pierce). We biotinylated rPR3 using a commercial biotinylation kit (Lightning-Link Rapid Biotin Conjugation Kit, Innova Biosciences), as previously published (18).

### PR3-reactive B cell detection and flow cytometry analysis.

PBMCs from trial participants with AAV and HCs were 10% DMSO cryopreserved PBMCs. Cryopreserved PBMCs were stained, and PR3^+^ B cells and Tfh cells were detected by flow cytometry analysis.

For PR3^+^ B cell detection, cells were counted, and 1 × 10^6^ cells were incubated on ice for 20 minutes with rPR3-biotin and a cocktail of different antibodies (anti-CD19-APC-Alexa Fluor 700, catalog A78837, clone J3-119; Anti-Human IgD-APC, catalog B30651, clone IA6-2; anti-CD27-PC7, catalog A54823, clone 1A4CD27; CD38-PC5.5, catalog A70205, clone LS198.4.3; and anti-CD24-APC-Alexa Fluor 750, catalog B10738, clone ALB9; all from Beckman Coulter), washed 3 times, incubated for 15 minutes with streptavidin-FITC (catalog 554060, BD Biosciences), washed, and fixed. For each experiment, unstained cells as well as single color controls were included. This customized flow cytometry assay, based on recombinant (r)PR3-FITC staining to identify the PR3-reactive B cells, was developed and validated by using anti-PR3 and anti-human neutrophil elastase antibody-producing hybridoma cell lines as positive and negative controls, as previously described in detail by our group (18).

For Tfh cell detection, cells were counted, and 1 × 10^6^ cells were stained with antibodies against human CD4 (clone RM4-5, Biolegend), CD45RO (Biolegend), PD-1 (clone 29F.1A12, Biolegend), and CXCR5 (clone 2G8, BD Biosciences).

Cell analysis was performed on a FACSCanto (BD Bioscience) or Attune NxT system (Life Technologies). FACS data were analyzed and graphed using KALUZA (Beckman Coulter) and FlowJo softwares.

The unsupervised clustering of flow cytometry data was performed using the SPADE algorithm (44) based on the level of expression of 6 markers on each B cell (PR3, CD19, CD27, IgD, CD38, and CD24). A total of 27 HCs, 105 participants with PR3-AAV, and 49 participants with MPO-AAV were analyzed. Briefly, after an initial downsampling, SPADE was performed using 200 nodes for the clustering. SPADE trees were initially visually investigated to identify and regroup nodes that exhibit similar phenotype. Then, data accompanying each SPADE tree (cluster abundance and MFI of each cluster for each sample) was downloaded from Cytobank, followed by hierarchical representation or testing for statistical significance.

### In vitro PR3-ANCA production by PBMCs and detection of PR3-specific B cells with ELISPOT.

PBMCs were cultured for 10 days to promote differentiation into antibody-secreting cells, after which PR3-ANCA secretion was measured in the supernatant by the EliA PR3S test (Thermo Fisher Scientific) on a Phadia ImmunocaCAP 250 analyzer (45).

Experiments were performed in duplicates in all trial participants with AAV and HCs. For each sample, 1 million PBMCs per well were cultured in 1 ml RPMI 1640 supplemented with 10% fetal calf serum and 50 mg/ml gentamicin (Gibco), in the presence of 3.2 μg/ml CpG-ODN 2006 (Hycult Biotechnology), 100 ng/ml BAFF (PreproTech), and 100 ng/ml IL-21 (PreproTech). PBMCs were cultured for 10 days to promote differentiation into antibody-secreting cells, after which PR3-ANCA secretion was measured in the supernatant by the EliA PR3S test on a Phadia ImmunocaCAP 250 analyzer (45)

To determine the ability of circulating PR3^+^ B cells to secrete in vitro immunoglobulins of different isotypes against PR3, we measured PR3-ANCA IgG and IgM by ELISPOT in peripheral blood of HCs. Briefly, enriched B cells from PBMCs of a HC were FACS sorted based on streptavidin expression to isolate PR3^+^ B cells and PR3^–^ B cells. The PR3^+^ B cells, PR3^–^ B cells, and total B cells were cultured for 24 hours with 1 μg/ml R848 (Mabtech) and 10 ng/ml recombinant human IL-2 (Mabtech). After 24 hours, prestimulated cells were added to the plate with 100 μl cell suspension/well (10,000 cells/well; 30,000 cells/well; and 15,000 cells/well for PR3^+^ B cells, PR3^–^ B cells, and total B cells, respectively). The ELISPOT was conducted according to the manufacturer’s instructions (Mabtech). This analysis could not be performed in the AAV trial participants, because the PBMCs required to obtain PR3^+^ B cells were in the order of 500 × 10^6^, much higher than that available from frozen PBMCs available for participants with AAV.

### Data availability.

Clinical data from the RAVE clinical trial are publicly available on the Immune Tolerance Network website (https://www.immunetolerance.org/researchers/trialshare). Experimental data are available upon reasonable request.

### Statistics.

Ordinal data are presented as *N* (%), and continuous data are presented as median (IQR) or mean ± SD. Groups were compared using parametric or nonparametric tests when appropriate; 2-tailed Student’s *t* test or 2-tailed Mann-Whitney test were used for continuous data. χ^2^ or Fisher’s test were used where appropriate for categorical data.

Multiple comparisons among more than 2 groups were performed with 1-way ANOVA or Kruskal-Wallis test, where appropriate. The multiple B cell maturation comparisons were analyzed by using mixed-effects modeling. Findings in all of the analyses were considered significant at *P* < 0.05 after adjustment for multiple comparisons by calculating the FDR as described by Benjamini and Hochberg (46). Spearman’s test was used to test correlations. The estimated distributions of time to remission were performed with the Kaplan-Meier method and the log-rank test. All statistical analyses were performed using GraphPad Prism software.

### Study approval.

All parent trial (RAVE trial) participants, from which the PBMC and serum samples used in the present study were derived, provided written informed consent for participation in the clinical trial as well as for the use of the biospecimens collected during the trial in future ancillary mechanistic studies. The trial protocol, including all provisions for the future use of biospecimens collected during the trial, was approved by the institutional review board of each participating trial site.

## Author contributions

AB, SH, US, and DC designed the study. AB, AMH, YMS, NC, WHA, PH, and DC acquired the data. AB, SH, NC, US, and DC analyzed the data and drafted the manuscript. AB, SH, US, DC, AMH, YMS, NC, WHA, PH, TP, EMC, KMH, EWSC, PB, FCF, CAL, CGMK, PA Merkel, PA Monach, PS, RFS, JHS, GG, JS, and JOP interpreted the results, analyzed the manuscript critically for important intellectual content, and approved the final version.

## Supplementary Material

Supplemental data

Trial reporting checklists

ICMJE disclosure forms

## Figures and Tables

**Table 1:**
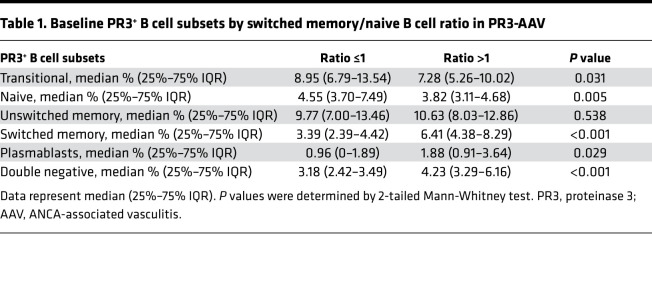
Baseline PR3^+^ B cell subsets by switched memory/naive B cell ratio in PR3-AAV

## Version 1 10/07/2021

In-Press Preview

## Version 2 11/22/2021

Electronic publication
