# Supplementary material for: Circulating autoreactive proteinase 3+ B cells and tolerance checkpoints in ANCA-associated vasculitis
Source: JCI Insight. 2021 Nov 22;6(22):e150999. doi: 10.1172/jci.insight.150999 (PMC8663783; doi:10.1172/jci.insight.150999)
Supplement: Supplemental data [file jciinsight-6-150999-s193.pdf]

SUPPLEMENTAL MATERIAL

SUPPLEMENTAL TABLES

Supplemental Table 1. Baseline features of patients with ANCA-associated vasculitis (AAV) and healthy controls (HCs) included in the study.

Characteristics	PR3-AAV (N=105)	MPO-AAV (N=49)	HCs (N=27)	p value
**Age at study entry,** media (SD)	48.7 (14.5)	59.0 (13.5)	53.9 (17.9)	**0.0004***
**Sex**				**0.0202***
**Male,** % (number)	59% (62)	37% (18)	41% (11)	
**Female,** % (number)	41% (43)	63% (31)	599% (16)	
**Ethnicity**				**0.3023**
**White,** % (number)	91% (96)	94% (46)	NA	
**Black,** % (number)	3% (3)	6% (3)	NA	
**Hispanic or Latino,** % (number)	5% (5)	-	NA	
**Asian,** % (number)	1% (1)	-	NA	
**Clinical Diagnosis,**% (number)				**<0.001**
GPA	97% (102)	33% (16)	-	-
MPA	3% (3)	65% (32)	-	-
**New Disease at enrollment** (vs relapsing disease), % (number)	42% (44)	78% (38)	-	**<0.001**
**Any granulomatous manifestation,** % (number)	76% (80)	35% (17)	-	**<0.001**
**Any capillaritis' manifestation,** % (number)	84% (88)	96% (47)	-	**0.0363**
**Any renal involvement,** % (number)	64% (67)	82% (40)	-	**0.0259**
**Alveolar hemorrhage,** % (number)	30% (32)	18% (9)		**0.1133**
**Glucocorticoid treatment at screening,** % (number)	54% (57)	59% (29)	-	0.6047
**Baseline BVAS/WG score,** mean (SD)	8.2 (3.3)	8.0 (3.1)	-	0.7351

**ESR** (SD), mm/1 hr, median [IQR]	33 [14.5; 56.5]	44 [15;77.5]	-	0.1414
**CRP** (SD), mg/L, median [IQR]	1.2 [0.35; 4.45]	1.2 [0.3; 3.025]	-	0.8521
**Baseline eGFR** (SD), mL/min/1.73 m^2, median [IQR]	92.0 [57.0; 127.6]	45.9 [30.1; 71.2]	-	**<0.001**
**ANCA levels (normal <20 IU)**				
MPO-ANCA	0.6 [0.3;1.1]	121 [56.9; 177]	-	**<0.001**
PR3-ANCA	258 [165.5; 346]	2.7 [1.4; 4.65]	-	**<0.001**
**Randomized Treatment group**				0.8629
Rituximab, % (number)	49% (52)	47% (23)	-	-
Cyclophosphamide/Azathioprine, % (number)	50% (53)	53% (26)	-	-

*One-way ANOVA: cut-off for p value interpretation after Bonferroni correction**: 0,0167**

Abbreviations: ANCA=anti-neutrophil cytoplasmic antibodies; BVAS/WG=Birmingham Vasculitis Activity Score for Wegener's Granulomatosis; CRP= C-reactive protein; ESR=erythrocyte sedimentation rate; eGFR=estimated glomerular filtration rate, by means the Modification of Diet in Renal Disease (MDRD) study equation; GPA=granulomatosis with polyangiitis; MPA-microscopic polyangiitis; MPO=myeloperoxidase; PR3=proteinase-3; SD=standard deviation; IQR=Interquartile range. P values are for the comparison of MPO-AAV and PR3-AAV groups.

***Footnotes**: Capillaritis was defined as the presence of one or more of the following BVAS/WG items: cutaneous purpura, scleritis, retinal hemorrhage or exudate, sensorineural deafness, hematuria, red blood cell casts on urinalysis or glomerulonephritis, increase in creatinine level, alveolar hemorrhage, mesenteric ischemia, sensory peripheral neuropathy, or motor mononeuritis multiplex. In contrast, BVAS/WG items reflecting underlying necrotizing granulomatous inflammation included mouth ulcers, retro-orbital mass/proptosis, bloody nasal discharge, sinus involvement, salivary gland enlargement, subglottic inflammation, conductive deafness, other major or minor ear/nose/throat involvement, pulmonary nodule/cavity, endobronchial involvement, meningitis, and cord lesion. Patients were considered to have renal disease if any renal item on the BVAS/WG (hematuria, red blood cell casts or glomerulonephritis, increase in creatinine level, or "other") was scored. A patient was categorized as having alveolar hemorrhage only if that item was scored on the BVAS/WG. All other BVAS/WG items cannot be clearly attributed to either necrotizing granulomatous inflammation or capillaritis and were, therefore, not considered to categorize the patient one way or another.*

Supplemental Table 2. Glucocorticoid treatment at screening (before sample collection). Results are represented as median (25-75% IQR).

Subset of disease	GC	No GC	P values
**PR3-AAV patients**			
Lymphocytes # (10^9cells/L)	1.12 (0.72,1.69)	1.16 (0.89,1.65)	0.591
Lymphocytes (% of PBMCs)	25.31 (15.66, 34.61)	28.93 (19.66, 40.19)	0.129
B cells (cells/µL)	123.69 (66.63, 182.64)	102.94 (68.98,217.63)	0.940
B cells (% of Lymphocytes)	10.61 (5.43, 16.22)	10.2 (5.32, 15.60)	0.589
$PR3^+$ B cells (cells/µL)	5.85 (3.33, 8.97)	4.69 (2.72, 10.12)	0.445
$PR3^+$ B cells (% of B cells)	4.82 (3.92, 6.30)	4.55 (3.99, 5.59)	0.246
Tfh (cells/mm^3)	20.95 (10.14, 48.62)	20.75 (8.44, 46.62)	0.599
Tfh (% of $CD4^+$ T cells)	3.58 (2.21, 4.88)	3.06 (1.83, 4.63)	0.144
**MPO-AAV patients**			
Lymphocytes # (10^9cells/L)	1.23 (0.93, 2.12)	1.25 (0.72, 2.33)	0.622
Lymphocytes (% of PBMCs)	25.57 (18.33, 34.64)	26.99 (19.89, 34.68)	0.504
B cells (cells/µL)	112.58 (56.22, 205.39)	118.92 (55.53, 236.65)	0.991
B cells (% of Lymphocytes)	8.43 (5.94, 17.87)	8.90 (6.07, 12.35)	1.000
$PR3^+$ B cells (cells/µL)	2.74 (2.02, 8.34)	3.68 (1.61, 9.66)	0.801
$PR3^+$ B cells (% of B cells)	3.13 (2.39, 5.04)	3.18 (2.72, 5.66)	0.569
Tfh (cells/mm^3)	27.52 (15.18, 48.50)	24.96 (10.21, 33.43)	0.411
Tfh (% of $CD4^+$ T cells)	4.05 (2.23, 5.59)	3.63 (2.69, 5.36)	0.954

ANCA=anti-neutrophil cytoplasmic antibodies; MPO=myeloperoxidase; PR3=proteinase-3; GC=glucocorticoids; IQR=Interquartile range.

P values were determined by 2-tailed Mann-Whitney test.

Supplemental Table 3 Phenotypic characterization of selected PR3$^+$ B cell clusters.

Cluster ID	Phenotype	Main Population
**Stable Clusters**		
CL 160	$IgD^+ CD24^{high} CD38^{high} CD27^-$	Transitional
CL 187	$IgD^+ CD24^+ CD38^+ CD27^-$	Bm2
CL 185	$IgD^+ CD24^+ CD38^{low} CD27^{low}$	Bm2 $CD27^{low}$
CL 93	$IgD^+ CD24^+ CD38^+ CD27^{low}$	Bm2 $CD27l^{ow}$
CL 57	$IgD^+ CD24^+ CD38^{low} CD27^{low}$	Bm2 $CD27^{low}$
CL 140	$IgD^+ CD24^+ CD38^{low} CD27^+$	UnSW 1
**Variable Clusters**		
CL 104	$IgD^- CD24^+ CD38^+ CD27^+$	PSW $CD38^+$
CL 132	$IgD^- CD24^{low} CD38^+ CD27^+$	PSW $CD38^+$
CL 155	$IgD^- CD24^- CD38^{++} CD27^{++}$	PB
CL 129	$IgD^- CD24^+ CD38^- CD27^-$	DN $CD38^-$
CL 186	$IgD^- CD24^+ CD38^+ CD27^-$	DN $CD38^+$

SUPPLEMENTARY FIGURES

Supplemental Figure 1. B cells and distribution of B cells among different subsets in HCs, MPO-AAV and PR3-AAV. Lymphocyte count (A), B cell count (B), and distribution of B cell count among the different B cell subsets (C). An explanatory example of the 200 B cell clusters obtained with SPADE (Spanning-tree Progression Analysis of Density-normalized Events) in a PR3-AAV patient (D). Heat Map of the 181 patients (*x axis*, HCs in red, MPO-AAV in green and PR3-AAV in blue) showing the different expression of each one of the 200 B cell clusters (*y axis*) (E). Principal component analysis of the B cell clusters representing HC subjects, MPO-AAV and PR3-AAV participants (F). Data are represented as median (25-75% IQR). Multiple comparisons between more than 2 groups were performed with Kruskal Wallis test and P values in the figures are indicated as * $p < 0.05$, ** $p < 0.01$, *** $p < 0.001$ after correction for FDR with Benjamini and Hochberg test.

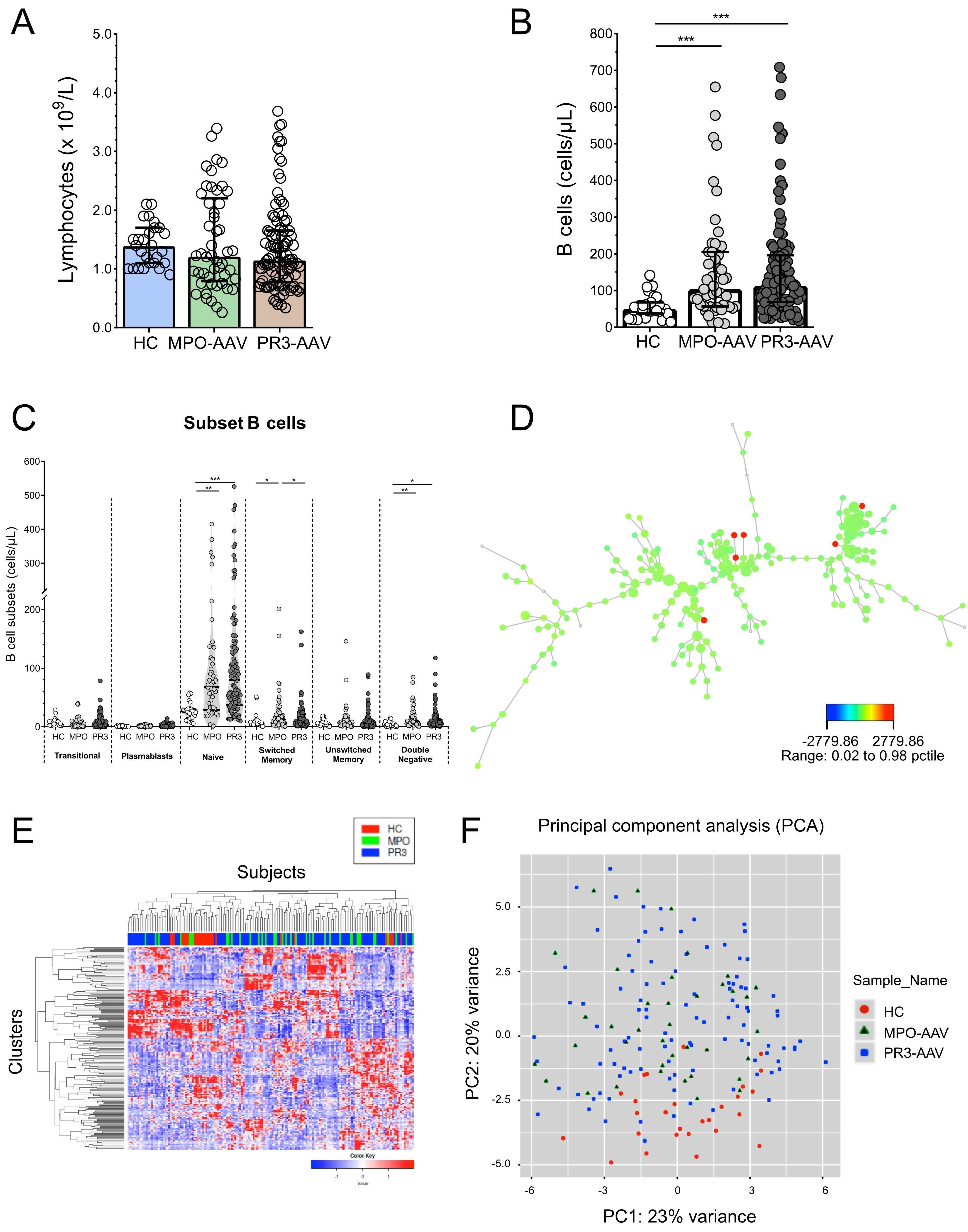
A
Lymphocytes (x 10^9/L)
5.0
4.0
3.0
2.0
1.0
0.0
HC
MPO-AAV
PR3-AAV
B
B cells (cells/μL)
800
700
600
500
400
300
200
100
0
***
***
HC
MPO-AAV
PR3-AAV
C
Subset B cells
B cell subsets (cells/μL)
600
500
400
300
200
100
0
HC MPO PR3
Transitional
Plasmablasts
Naive
Switched Memory
Unswitched Memory
Double Negative
D
-2779.86
2779.86
Range: 0.02 to 0.98 pctile
E
HC
MPO
PR3
Subjects
Clusters
Color Key
F
Principal component analysis (PCA)
PC2: 20% variance
PC1: 23% variance
5.0
2.5
0.0
-2.5
-5.0
-6
-3
0
3
6
Sample_Name
HC
MPO-AAV
PR3-AAV

Supplemental Figure 2. Circulating PR3$^+$ B cells and PR3-ANCA production. Purification of PR3$^+$ B cells and PR3$^-$ B cells for the ELIspot analysis in HCs (A). PBMCs isolated from a healthy subject were enriched for B cells by anti-CD19 antibody-linked magnetic bead selection. The B cell enriched fraction was FACS-sorted based on streptavidin expression to isolate PR3$^+$ B cells and PR3$^-$ B cells. Purity was 99% for PR3$^+$ B cells and 96% PR3$^-$ B cells, respectively.

Despite the production of total IgG and IgM by B cells (shown in B, left), PR3-ANCA IgM, but not PR3-ANCA IgG, can be secreted by PR3$^+$ B cells (shown in B, central).

Circulating (*in vivo*) PR3-ANCA IgG in PR3-AAV participants by ELISA (C).

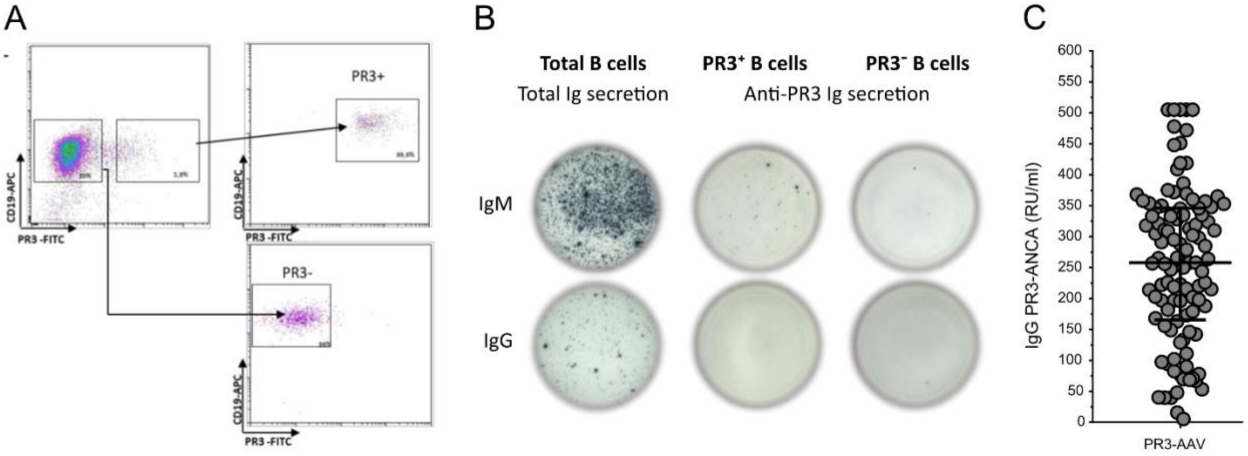

Supplemental Figure 3. Representative B cell subset plots of a HC subject, a MPO-AAV patient and a PR3-AAV patient. PR3$^+$ B cells recognizing nuclear antigens were identified within each B cell subset in AAV and HC.

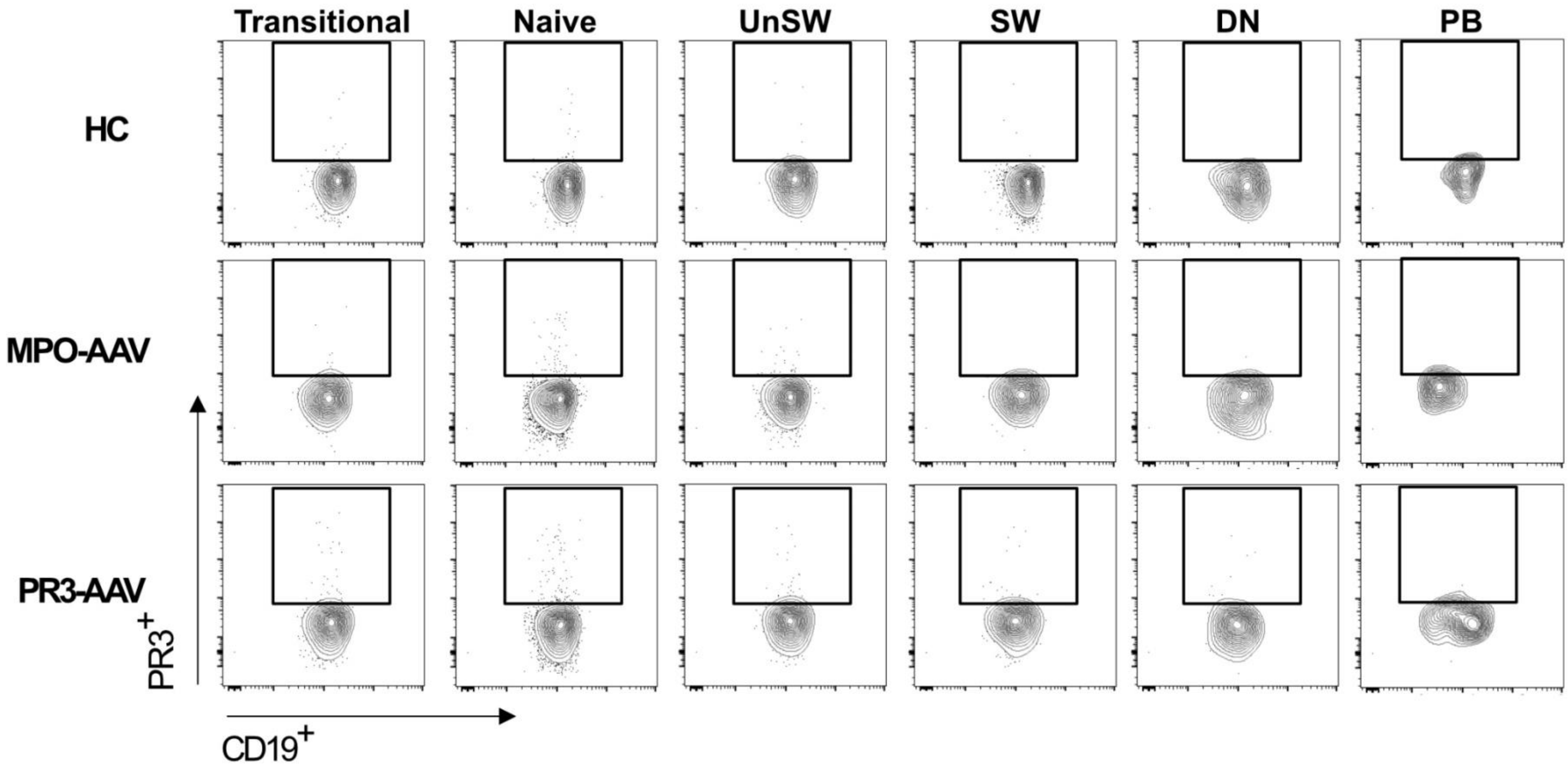

Supplemental Figure 4. Unsupervised clustering of circulating PR3+ B cells. Mean fluorescence intensity (MFI) of 2 out of 6 clusters with stable expression of PR3 across the samples (CL185 and CL57) (A), the others are represented in Figure 5. Data represent mean ± SD in histograms. $PR3^{high}$ B cell distribution in the B cell subsets (B); frequencies of $PR3^{high}$ in transitional and naïve B cells are higher in AAV participants compared to HCs, but only the frequencies of $PR3^{high}$ in DN B cells were higher in PR3-AAV participants compared to both MPO-AAV participants and HCs. Data represent median (25-75% IQR) in violin plots, or mean ± SD in histograms. Multiple comparisons between more than 2 groups were performed with one-way ANOVA or Kruskal-Wallis test, where appropriate. P values in the figures are indicated as * $p < 0.05$, ** $p < 0.01$, *** $p < 0.001$ after adjustment for FDR as described by Benjamini and Hochberg.

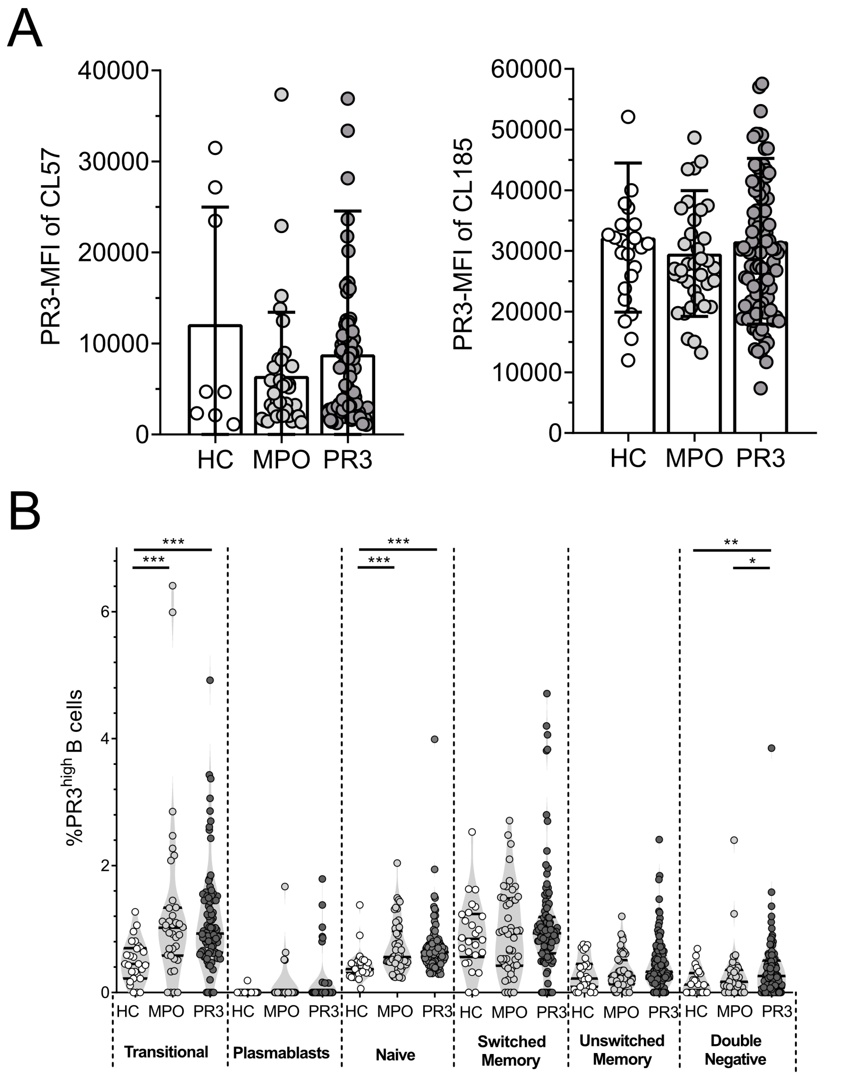
A
PR3-MFI of CL57
40000
30000
20000
10000
0
HC
MPO
PR3
PR3-MFI of CL185
60000
50000
40000
30000
20000
10000
0
HC
MPO
PR3
B
%PR3^{high} B cells
6
4
2
0
***
***
***
***
**
*
HC MPO PR3
Transitional
Plasmablasts
Naive
Switched Memory
Unswitched Memory
Double Negative

Supplemental Figure 5. Determinants of $PR3^+$ B cell maturation. Frequency of AAV participants and HC subjects with a positive PR3+ B cell memory/naïve ratio (A). Levels of BAFF, CXCL13, IL-6 and in MPO-AAV and PR3-AAV participants (B). Gating strategy used for TFH identification (the reference population for the frequency is $CD4^+CD45RO^+CXCR5^+PD\text{-}1^+$) (C), TFH cell count and frequency in HCs, MPO-AAV and PR3-subects (D), and association of TFH higher levels with ratio ≤ 1 in PR3-AAV participants (E). Each point represents the frequency in an individual subject; horizontal lines show the median with 25-75% IQR**.** P values were determined by 2-tailed Mann-Whitney test. Multiple comparisons between more than 2 groups were performed with Kruskal-Wallis test.P values in the figures are indicated as * $p < 0.05$, ** $p < 0.01$, *** $p < 0.001$ after adjustment for FDR as described by Benjamini and Hochberg.

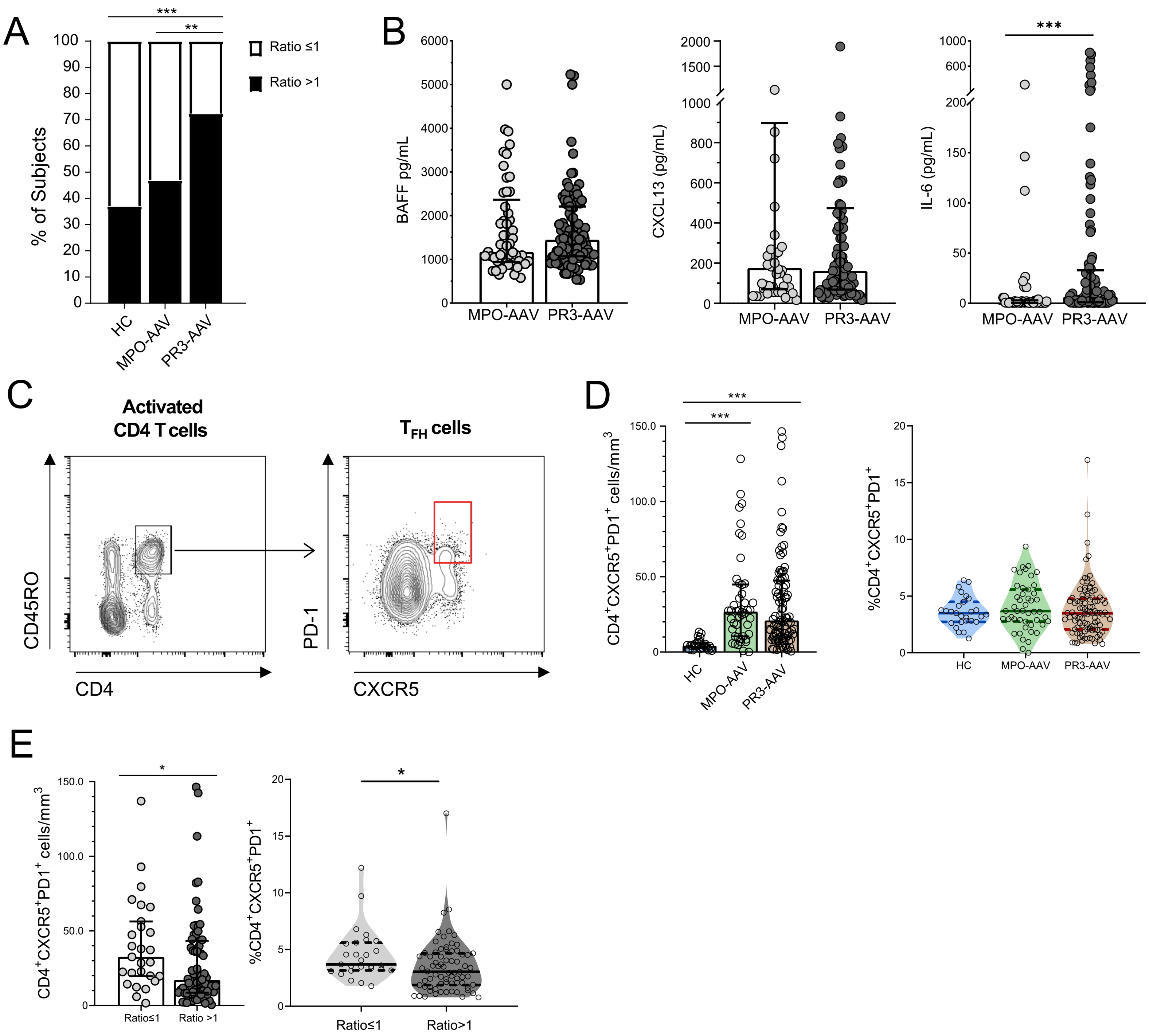

A
% of Subjects
Ratio ≤1
Ratio >1
HC
MPO-AAV
PR3-AAV
***
**
B
BAFF pg/mL
CXCL13 (pg/mL)
IL-6 (pg/mL)
MPO-AAV
PR3-AAV
C
Activated
CD4 T cells
T_{FH} cells
CD45RO
CD4
PD-1
CXCR5
D
$CD4^+CXCR5^+PD1^+$ cells/mm^3
$\%CD4^+CXCR5^+PD1^+$
E
Ratio≤1
Ratio >1
*

Supplemental Figure 6. SW memory/naïve PR3^{+} B cell ratio and clinical manifestations in PR3-AAV patients. In PR3-AAV patients (n=105), SW memory/naïve PR3^{+} B cell ratio did not correlate with Body Mass Index (BMI) (A), relapsing versus new diagnosis (B), previous use of glucocorticoids (C), the presence of manifestations reflecting granulomatous disease only (D) or capillaritis only (E), as assessed by the BVAS/WG. The ratio did not correlate with disease activity as assessed by BVAS/WG (E). Time to first remission (BVAS/WG=0, any prednisone dose) (F) or time to complete response (BVAS/WG=0, prednisone dose≤10mg/day) (G) are represented. When evaluating associations with remission, the subjects that underwent cross over (n=7) or experienced early treatment failure (n=6) during the trial time were excluded from the analysis. P values in the figures are indicated as * $p < 0.05$, ** $p < 0.01$, *** $p < 0.001$

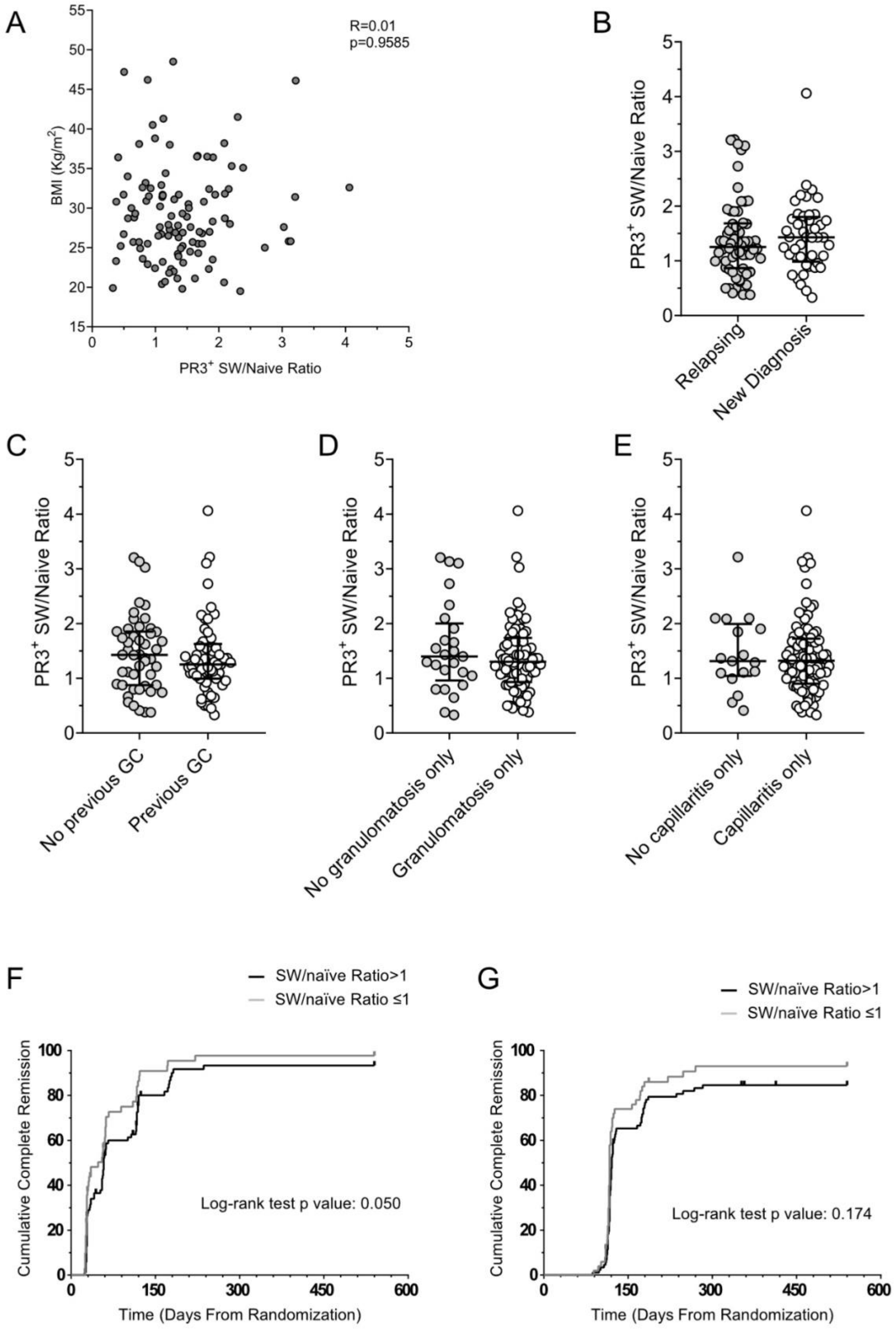
A
R=0.01
p=0.9585
BMI (Kg/m²)
PR3⁺ SW/Naive Ratio
B
PR3⁺ SW/Naive Ratio
Relapsing
New Diagnosis
C
PR3⁺ SW/Naive Ratio
No previous GC
Previous GC
D
PR3⁺ SW/Naive Ratio
No granulomatosis only
Granulomatosis only
E
PR3⁺ SW/Naive Ratio
No capillaritis only
Capillaritis only
F
SW/naïve Ratio>1
SW/naïve Ratio ≤1
Cumulative Complete Remission
Log-rank test p value: 0.050
Time (Days From Randomization)
G
SW/naïve Ratio>1
SW/naïve Ratio ≤1
Cumulative Complete Remission
Log-rank test p value: 0.174
Time (Days From Randomization)

Supplemental Figure 7. Frequency of PR3$^+$ B cells and clinical manifestations in PR3-AAV participants. In PR3-AAV patients (n=105), frequency of PR3$^+$ B cells did not show any meaningful correlation with age (A), sex (B), BMI (C), BVAS/WG (D), CRP (E), ESR (F), renal involvement (G) or creatinine clearance (H), relapsing versus new diagnosis (I), previous use of glucocorticoids (L), the presence manifestations reflecting granulomatous disease only (M) or capillaritis only (N), as assessed by the BVAS/WG. P values in the figures are indicated as * $p < 0.05$, ** $p < 0.01$, *** $p < 0.001$

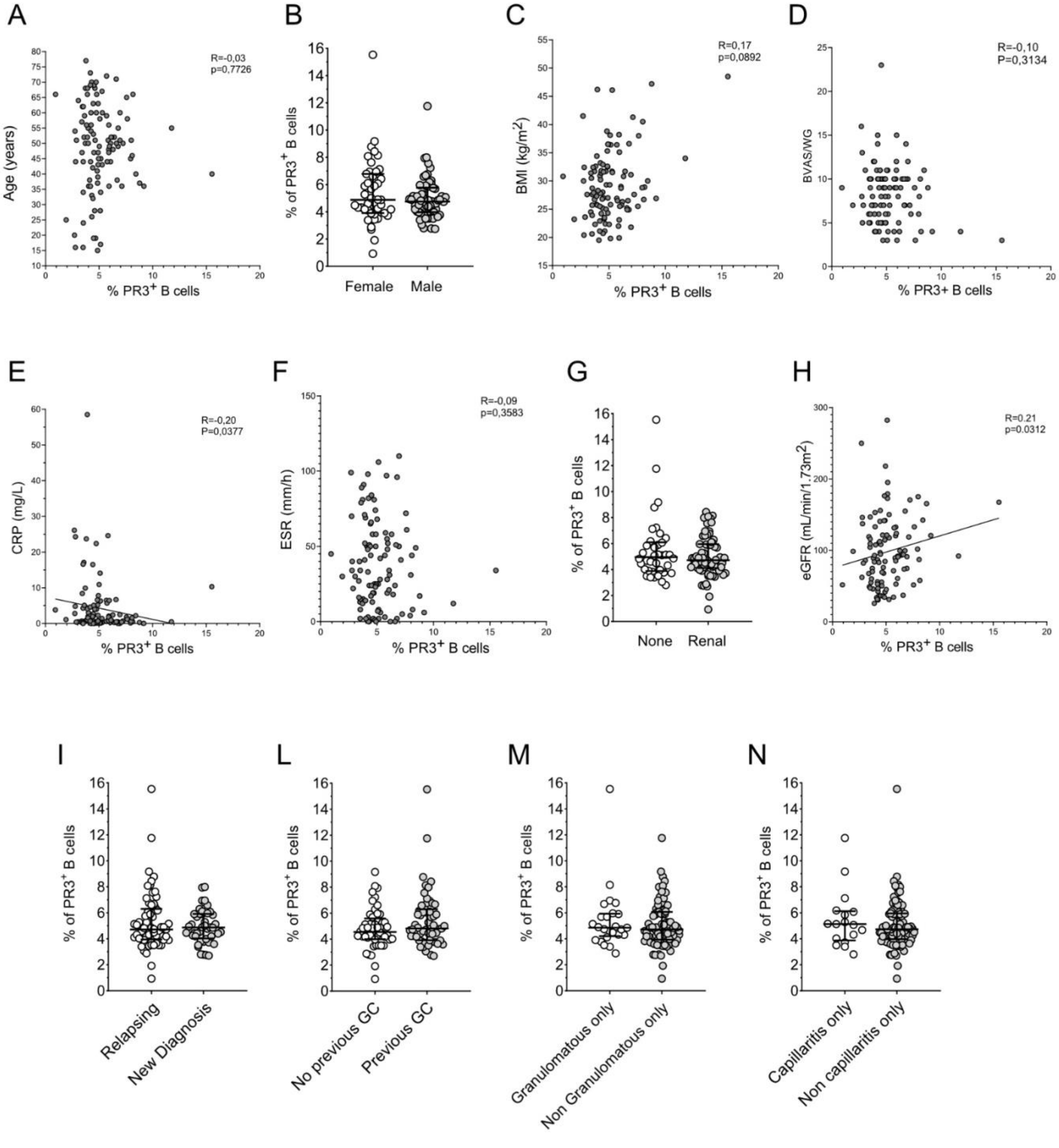

A
Age (years)
R=-0,03
p=0,7726
% PR3⁺ B cells
B
% of PR3⁺ B cells
Female
Male
C
BMI (kg/m²)
R=0,17
p=0,0892
% PR3⁺ B cells
D
BVAS/WG
R=-0,10
P=0,3134
% PR3+ B cells
E
CRP (mg/L)
R=-0,20
P=0,0377
% PR3⁺ B cells
F
ESR (mm/h)
R=-0,09
p=0,3583
% PR3⁺ B cells
G
% of PR3⁺ B cells
None
Renal
H
eGFR (mL/min/1.73m²)
R=0.21
p=0.0312
% PR3⁺ B cells
I
% of PR3⁺ B cells
Relapsing
New Diagnosis
L
% of PR3⁺ B cells
No previous GC
Previous GC
M
% of PR3⁺ B cells
Granulomatous only
Non Granulomatous only
N
% of PR3⁺ B cells
Capillaritis only
Non capillaritis only

SUPPLEMENTAL ACKNOWLEDGMENTS

RAVE-ITN Research Group:
Co-Protocol Chairs: Ulrich Specks, M.D. (Mayo Clinic) John H. Stone, M.D., M.P.H. (Massachusetts General Hospital)

Mayo Clinic: Ulrich Specks, M.D. Steven R. Ytterberg, M.D. Fernando C. Fervenza, M.D. Karina A. Keogh, M.B., B.Ch. Tobias Peikert, M.D. Jason M. Golbin, D.O. Lorenzo Klein, M.D. Kathleen Mieras, C.C.R.P. Cynthia Beinhorn Susan Fisher, R.N. Mary Lou Clawson, R.N. Sharon Bendel, R.N. Amber M. Hummel, Mayo Clinic Eisenberg Research Pharmacy.

Boston University: Peter A. Merkel, M.D., M.P.H. Eugene Y. Kissin, M.D. Paul A. Monach, M.D., Ph.D. Manuella R. Clark-Cotton, M.A. Carol A. McAlear, M.S. Jessica L. Pettit Maureen B. Sutton, M.P.H. Russell L. Widom, Ph.D. Giuseppina A. Farina, M.D., Ph.D. Michael J. DiMarzio Sharlene P. Johnson, M.A.T.

Johns Hopkins University: Philip Seo, M.D., M.H.S. John H. Stone, M.D., M.P.H. David Hellmann, M.D. Duvuru Geetha, M.D. Assil Saleh, M.D., M.P.H. Peter Wung, M.D. Lourdes P. Sejismundo, R.N., B.S.N. Charlotte Humphrey, R.N., B.S.N. Matthew Marriott, P.A.-C. Yavette Goldsborough, Alexander Pinachos Karen Gauss, R.N., M.L.A. Linda King, R.N.

Cleveland Clinic: Foundation Carol A. Langford, M.D., M.H.S. Gary S. Hoffman, M.D. Rula A. Hajj-Ali, M.D. John J. Carey, M.D., M.S. Eamonn S. Molloy, M.D., M.S. Curry L. Koening, M.D., M.S. Debora Bork, M.F.A. Tiffany M. Clark, M.S.N., C.N.P. Katherine A. Tuthill, M.S.N., C.N.P. Teresa Markle John Petrich, R.Ph., M.S.

Hospital for Special Surgery: Robert Spiera, M.D. Deborah R. Alpert, M.D., Ph.D. Stephen J. DiMartino, M.D., Ph.D. Jessica K. Gordon, M.D. Neal K. Moskowitz, M.D., Ph.D. Kyriakos A. Kirou, M.D. Jonathan Samuels, M.D. Stacey A. Kloiber, R.N. Elvedin Julevic Margaret O'Donohue, R.N. Avni Patel, Pharm.D.

University of Groningen: Cees Kallenberg, M.D., Ph.D. Coen Stegeman, M.D., Ph.D. Peter Rasker, R.N. Katinka Mulder Pieter Limburg, Ph.D. Jos Kosterink, Pharm.D.

Duke University: E. William St. Clair, M.D. Nancy B. Allen, M.D. Edna Scarlett, R.N. Martin Tochacek, Ph.D.

University of Alabama at Birmingham: Anthony Turkiewicz, M.D. Barri J. Fessler, M.D. Winn Chatham, M.D., Anita Turner, R.N.

Coordinating Centers:
Rho: David Ikle, Ph.D. Alice Lail, M.S.. Brett Jepson, M.S.
PPD: Wei Wu, Ph.D. Tammy D'Lugin Cathy Jacob
National Institute of Allergy and Infectious Diseases: Lisa Viviano, R.N.* Linna Ding, M.D., Ph.D. Steven Adah, Ph.D. James McNamara, M.D.
Immune Tolerance Network: Nadia Tchao, M.D. Mark Mueller, B.S., C.C.R.P.** Kasia Bourcier, Ph.D.** Deborah J. Phippard, Ph.D. Adam L. Asare, Ph.D. Noha Lim, Ph.D.

Peter Sayre, M.D. Vicki-Seyfert Margolis, Ph.D.*** Patti Tosta, R.N. Nancy B. Skeeter Claire L. Anderson Adelaide N. Archampong

*Current employer: National Institutes of Heart, Lung and Blood Diseases. **Current employer: National Institutes of Allergy and Infectious Diseases. ***Current employer: The U.S. Food and Drug Administration. The views presented in this article do not necessarily reflect those of the Food and Drug Administration.
